# A Proposal for IoT Dynamic Routes Selection Based on Contextual Information

**DOI:** 10.3390/s18020353

**Published:** 2018-01-26

**Authors:** Harilton da Silva Araújo, Raimir Holanda Filho, Joel J. P. C. Rodrigues, Ricardo de A. L. Rabelo, Natanael de C. Sousa, José C. C. L. S. Filho, José V. V. Sobral

**Affiliations:** 1 Programa de Pós-Graduação em Informática Aplicada (PPGIA), University of Fortaleza (UNIFOR), Av. Washington Soares, 1321, Edson Queiroz, 60.811-905 Fortaleza-CE, Brazil; harilton@edu.unifor.br (H.d.S.A.); raimir@unifor.br (R.H.F.); 2 Instituto de Telecomunicações, Av. Rovisco Pais, 1, 1049-001 Lisboa, Portugal; jose.sobral@it.ubi.pt; 3 Department of Computer Science, University Estacio of Sá, Av. Expedicionários, 790, São João, 64.046-700 Teresina-PI, Brazil; 4 National Institute of Telecommunications (Inatel), Av. João de Camargo, 510-Centro, 37540-000 Santa Rita do Sapucaí-MG, Brazil; 5 International Laboratory “Technosphere Safety”, ITMO University, 49 Kronverksky Pr., St. Petersburg 197101, Russia; 6 Department of Computing, Federal University of Piauí (UFPI), Department of Computing, 64.049-550 Teresina-PI, Brazil; ricardoalr@ufpi.edu.br (R.d.A.L.R.); csousa.natanael@gmail.com (N.d.C.S.); jcarloslimafilho@gmail.com (J.C.C.L.S.F.); 7 Departamento de Informática, Universidade da Beira Interior, Rua Marquês D’Ávila e Bolama, 6201-001 Covilhã, Portugal; 8 Department of Education, Federal Institute of Maranhão (IFMA), Av. Getúlio Vargas, 4, Monte Castelo, 65030-005 São Luiz-MA, Brazil

**Keywords:** Internet of Things, routing, 6LowPAN, context-aware, objective function, fuzzy system

## Abstract

The Internet of Things (IoT) is based on interconnection of intelligent and addressable devices, allowing their autonomy and proactive behavior with Internet connectivity. Data dissemination in IoT usually depends on the application and requires context-aware routing protocols that must include auto-configuration features (which adapt the behavior of the network at runtime, based on context information). This paper proposes an approach for IoT route selection using fuzzy logic in order to attain the requirements of specific applications. In this case, fuzzy logic is used to translate in math terms the imprecise information expressed by a set of linguistic rules. For this purpose, four Objective Functions (OFs) are proposed for the Routing Protocol for Low Power and Loss Networks (RPL); such OFs are dynamically selected based on context information. The aforementioned OFs are generated from the fusion of the following metrics: Expected Transmission Count (ETX), Number of Hops (NH) and Energy Consumed (EC). The experiments performed through simulation, associated with the statistical data analysis, conclude that this proposal provides high reliability by successfully delivering nearly 100% of data packets, low delay for data delivery and increase in QoS. In addition, an 30% improvement is attained in the network life time when using one of proposed objective function, keeping the devices alive for longer duration.

## 1. Introduction

Internet of Things (IoT) is referred to as the interconnection of physical objects that have an IP address for Internet connectivity. A huge number of smart devices are interconnected to the Internet today. There is an exponential growth in the number of smart devices interconnected through the mobile Internet [1]. IoT can be used in architectures, such as the proposal presented in [2], which represents an architecture for two-way communication between smart utility meters and utility companies. Is a wireless sensor network that provides communication for metering devices in the neighborhood area of a smart grid. Another point that is addressed in IoT is mobility, which is a fundamental issue related to the detection of mobile nodes movement and the provision of better links through an efficient routes selection [3]. Many contributions are evidenced in the literature in the IoT topic, such as the following: study of mechanisms for the topology control [4], location and mobility in Wireless Sensor Networks (WSNs) [5] and routing based on objective functions [6,7,8]. It is considered also as pervasive presence of devices variety, such as sensors, Radio Frequency Identification (RFID) tags and smartphones, among other devices which can interact with each other for a common purpose [9]. In a scenario of IoT, plurality is increasing and forecasts indicate that over 40 billion devices will be connected until 2020 [2], allowing the emergence of an infinity of new applications, such as for smart cities, healthcare, smart houses, industry, agriculture, etc.

Among the most promising technologies for the IoT paradigm, RFID and Wireless Sensor Networks (WSNs) are the most common and appropriate [9,10]. WSNs have limitations on the identification of a person or object in some types of applications. However, unlike WSNs, RFID systems are unable to sense data from the place in which they are used, such as humidity, temperature and pressure that are provided by sensors. This is an indication that IoT, by means of the integration between the RFID and WSN technologies, maximizes the benefits, thus opening up new perspectives for applications that consider context information, such as temperature monitoring in remote areas and air quality of a specific city or region, vehicle control flows, among others.

The context usually refers to the collected information regarding a given situation and may include also the location. Moreover, it can also cover different information used to characterize entities involved in the interaction between the user and the application. According to [11], context-sensitive systems are able to adapt their behavior to current conditions with no explicit intervention from users.

In IoT, an amount of information, such as features of the device itself, of the environment which surrounds it and its capacity, can be used as a source of context information. Many papers available in literature are converging in efforts to address issues involving context sensitivity in IoT, especially at the route selection stage [12,13,14]. At this stage, devices (which have resource constraints) process contextual information locally in order to select the path that best meets the requirements of a given application. Such feature requires context-sensitive routing protocols for the fulfillment of a number of challenges during messages exchange, including shorter delay, greater reliability on data transmission and minimal power consumption. Based on these challenges, this paper proposes an improvement for the RPL protocol [15] that considers the creation of four new Objective Functions (OFs) that enable the devices to select the parent node (default route) based on the context information obtained from the application. The use of proposed OFs occurs in the process of establishing routes in order to meet the context of the applications. This is an optimization problem, which seeks to maximize the reliability of data packet delivery, minimize the delay and increase QoS and network lifetime by using one or more of the objective functions proposed in this work. The objective functions meet a set of constraints (traditionally, constraints are expressed by algebraic equations). In this way, the objective function is used as a means of quantifying and qualifying the potential solutions of the problem in question. Then, the main contributions of this work are the following: (i) a proposal of creation of four new Objective Functions (OFs) that are used dynamically and at run time and (ii) a Fuzzy System-Based Route Classifier that enable the devices to establish routes high quality order to meet the context of the applications specific. 

The rest of the paper is organized as follows. Section 2 sets out the related work on the topic and Section 3 describes the proposed improvement on the RPL protocol. The performance assessment of the proposed approach is discussed in Section 4, followed by the conclusions and research directions in Section 5.

## 2. Related Work

According to [16], RPL makes use of metrics specified in the RFC 6551 which are suitable for IPv6 over Low Power Wireless Personal Area Networks (6LoWPAN) environments, considering the number of hops; latency; delivery ratio, node energy; throughput; level of link quality; and transmission reliability.

In [17], a standard objective function, called Objective Function Zero (F0), was proposed for RPL. It was designed to enable interoperability among different implementations of RPL. The operation of OF0 is simplified and does not use any routing metrics for the definition of the rank (position in the routing tree). A device chooses its preferred node considering the neighbor within those that presents the lowest rating. 

The Minimum Rank with Hysteresis Objective Function (MRHOF) it is presented in [6]. This OF is only suitable for the metrics specified in RFC 6719. The selection of the favorite “parents” is made taking into account the adopted metrics, where the routes that minimize the cost associated with the metrics are preferable. As standard, MRHOF uses the Expected Transmission Count (ETX) metric, from [14], which assesses the quality of the link and aims to maximize the leakage of data. The ETX is defined according to Equation (1).

(1) $$\mathrm{ETX}\;=\;\frac{1}{D_{f}\cdot D_{r}}$$

where:

- $D_{f}$ is the probability of the package being received by the neighbor; and
- $D_{r}$ is the probability of the ACK being received successfully.

The authors of [18] proposed a context-aware approach that changes the functioning of a sensor node based on the phenomena collected from the environment, such as temperature, humidity, pressure, etc. This contribution called Situation-Aware Adaptation Approach for Energy Conservation in Wireless Sensor Network (SA-A-WSN), aims to monitoring the way the sensor node works in the environment in order to reduce the network’s power consumption. 

According to [13], context-sensitive computing has been gaining more market and relevance since it is currently being considered an important component of IoT, mainly because context-sensitive systems are able to adapt their behavior to current conditions with no explicit intervention from users.

A context-sensitive objective function, called Context Aware Objective Function (CAOF), proposed by [14], is based on residual resources and on changing the sensor node’s status over time. The function proposed by [15] performs a weighted sum of the following metrics: degree of the node connectivity, level of battery power and position of the node in the routing tree regarding the parent node. The ultimate goal of the function proposed in [15] is to find a probability of delivery to each sensor node.

The use of fuzzy logic to calculate the objective function for RPL protocol is proposed in [19]. The authors describe that the objective function is the component used to select the paths by identifying a parent node among many existing nodes. The objective function, called QoS-aware fuzzy logic objective function (OF-FL), associates parameters with linguistic variables which are combined with fuzzy rules in order to identify the route to be selected. The parameters considered in OF-FL are hop numbers, end-to-end delay, packet loss rates and default route change rate.

In [20], a Scalable Context-Aware Objective Function (SCAOF) is proposed. It adapts the RPL protocol to the environmental monitoring within the area of agriculture with a scalable context. The performance of the SCAOF was assessed both through simulation and field testing. The experimental results obtained confirm that SCAOF can extend the network’s service life and improve the Quality of Service (QoS) in the different agriculture-oriented simulation scenarios. 

The work included in [6,7,8] represents recent research work considering objective functions in the route selection process. In [6], the performance of two RPL objective functions (the Minimum Classification with Hysteresis Objective Function (MRHOF) and the Function Zero Objective (OFO)) are analyzed. It is observed that MRHOF offers better performance than OF0 in terms of network quality. The MRHOF is suitable for use in sensor network that require data delivery in a reliable network. In turn, OF0 is suitable for use in sensors network that require fast network connection training and low power consumption.

A Smart Energy Efficient Objective Function (SEEOF) is proposed in [7]. It was designed to create an IPv6 mesh topology for IoT based on smart metering applications. The SEEOF is based on RPL and it is designed to use energy efficiently and extend the network lifetime. It mainly takes into account the node energy. The energy consumption in RPL using SEEOF was compared with MRHOF [6]. Simulation results show that an improvement up to 27% is attained in the network life time when using the proposed objective function. More important, the results show that SEEOF balances the energy consumption more uniformly among the battery powered devices keeping them alive for longer duration. 

In [8], an Energy Efficient and Path Reliability Aware Objective Function (ERAOF) for IoT applications that requires energy efficiency and reliability in data transmission is proposed. The ERAOF is based on the composition of the following metrics: node energy and link quality. Different from the proposal presented in [7], ERAOF considers not only the energy consumed but also the quality of the link in the process of route selection. This characteristic motivates its inclusion in the comparative analysis performed in this work.

The proposal of this work was based mainly on the works presented in [7,8,14,19,20]. These works motivated the authors to propose a new approach for five main reasons: (i) to use appropriate metrics for 6LoWPAN environments; (ii) to perform a weighted average of the metrics to provide quality message transmission through a sensor network without wire; (iii) to use the fuzzy logic objective function; (iv) to use an energy efficient objective function; and (v) to use Context-Aware Objective Function. The proposed approach is presented in the next section.

## 3. Proposed Approach

In this paper, we propose an adaptation of the Routing Protocol for Low-power and Lossy-networks (RPL)—(RFC 6550) protocol. The main purpose of this proposal is to optimize routing in order to meet the context requirements of specific applications.

### 3.1. The RPL Protocol

RPL is a network layer protocol and specifically designed to be used along with 6LoWPAN. It operates under several mechanisms of the connection layer, including the IEEE 802.15.4 MAC and PHY layers [17]. Please refer to Table 1 for a description of the symbols used in the remainder of this section.

An IoT scenario with RPL can be modeled as a graph *G =* (*N*, *L*), where *N* is the set of *n* network nodes (excluding the root node) and *L* is the set of connections that link such nodes. The graph *G* represents the creation of the network topology including the created routes and sent messages. For each *ni* ∈ *N* (*i* = 1, 2, ..., *n*) there is a set *A* that contains nodes enabled to be candidates to become the parent node of another node. This process is performed based on the rank of each node. The rank is a number that identifies the position of a node in the topology relative to the other nodes and to the root of the graph. The use of ranks makes it possible for a node to differentiate its position from the parent nodes and sibling nodes. It consolidates a mechanism where a node decides its parent node according to the smaller rank, which is defined as an integer representing the individual position of the node within the graph *G*. This parameter increases its value as it descends in the network topology. The nodes involved in the creation of this topology are represented by Expressions (2) and (3):

(2) N = {n1, n2, …, nn}

(3) A(ni) = {a1, a2, …, am}

where:

- *N* is the set of nodes of a network;
- *A*(*ni*) is the set of progenitor nodes of a given node.

Considering that *A* is a subset of the total number of nodes, then, *A*(*ni*) ⊂ *N*. The root node represented as *ns*, is the only node from the network which has no list of candidates because sent messages only downward direction; that means its subset is empty, Equation (4):

*A*(*ns*) = ∅ (4)

For each *ni* node there is a set of *m* connections *l* equal to the number of candidate nodes, as demonstrated in Equation (5). This means that each node has a number of connections (edges) corresponding to the number of nodes that are candidates to be progenitors. For example, if a node has a connection to six other nodes, there are six candidates to be progenitors. In a set *L* there is an *l* connection that belongs to *L*(*ni*). Therefore, the conclusion is that *aj* belongs to *A*(*ni*), where *aj* is the candidate node selected to be the parent node of *ni*.

*L*(*ni*) = {*l*1, *l*2, …, *l*n} (5)

In Equation (6), each connection (represented by “*l*”) has a rank, which equals the rank calculated in the *ni* node in relation to a selected parent node *aj* (*j* = 1, 2, …, *m*). The rank is obtained based on the position of a node in the topology regarding the other network nodes. In this case, a node decides who is its parent node according to the lowest rank.

*l*(*ni*, *aj*) = *rank* (6)

Equation (7) must be followed because a candidate node can be selected as a parent node. In this case, when the rank of a node *ap* (receiver node) is greater than the rank of *aj* (sender), the *ap* accepts *aj* as its parent node, creating through it a path to *ns* (root node). Otherwise, it rejects the transmitter as its parent node. 

*l*(*ni*, *ap*) > *l*(*ni*, *aj*) ∀*aj* ∈ *l* (7)

In conclusion, the construction of the network for each *ni* node can be defined as follows:

*C =* {∀*ni* ∈ *N*, ∋ *ap* ∈ *A*(*ni*)*:l*(*ni*, *ap*) *> l*(*ni*, *aj*) ∀*aj* ∈ *l*} (8)

RPL protocol is implemented in 4 stages: (a) setup stage; (b) routes establishment stage; (c) data communication stage; and (d) path reconstruction stage.

At the route Setup stage, the RPL protocol (in its original version) uses the OF0 to enable the nodes to select the parent nodes (default route), based on information from the rank and on the number of hops of a given node for the sink, as described in Equation (9):

*R*(*N*) = *R*(*P*) + *rank__increase_* (9)

where:

- *R*(*N*), is the node new rank;
- *R*(*P*), is the preferred parent rank;
- *rank__increase_*, is a variation factor (delta) between the node and the parent rank.

### 3.2. Proposed Approach

In order to improve RPL protocol, four objective functions are proposed to be used at the routes establishment stage: DQCA-OF1, DQCA-OF2, DQCA-OF3 and DQCA-OF4. Each one of these objective functions is based on the fusion of metrics for 6LoWPAN environments, used by the RPL and described in RFC 6551. The context information is used as a parameter for selecting the one that best suits a given application, among the four available objective functions. 

The APPLICATIONS module represents the context of each application. The DATABASE module contains the objective functions which will be used for generating routes that meet the requirements of each application.

**Figure 1:**
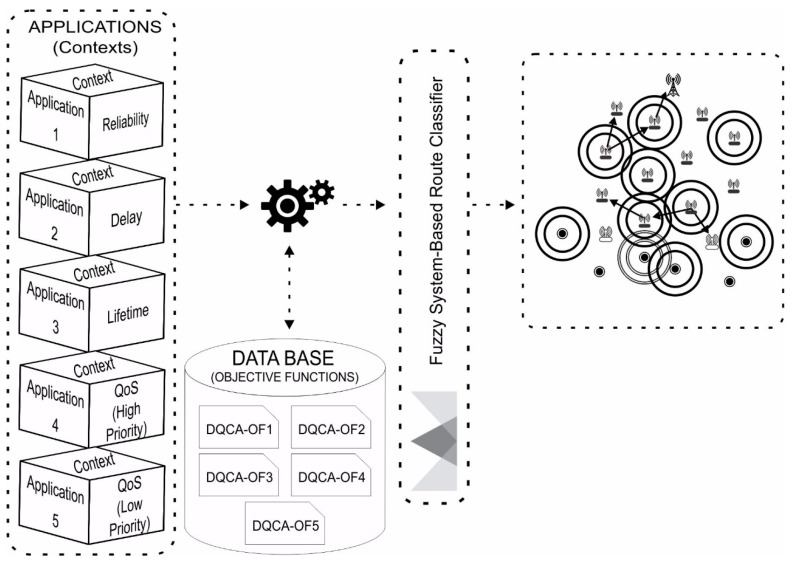
Functional model of the proposed approach.

Figure 1 illustrates the functioning of the proposed approach, which is based on the relationship among three nodes. 

#### 3.2.1. Objective Functions

Existing objective functions have constraints, since an objective function composed by a single routing metric presents advantages and limitations. A single metric present in the objective function may not fully satisfy the requirements required by the applications. For example, while the number of jumps allows choosing the shortest path, this can lead to the failure of one or more nodes due to power consumption because the battery level is not considered in the decision process. In addition, considering ETX as a single routing metric can lead to high latency in routing messages. When selecting routes with low ETX, the network becomes more reliable but does not reduce the latency during the message routing process. Thus, the ETX metric alone is not suitable for real-time applications because it does not take into account the delivery time requirements of the messages. 

The fixed and permanent combination of two metrics is insufficient to efficiently meet all the requirements of the applications, since the requirement of each application may change eventually. In addition, considering two routing metrics it is possible to optimize routing performance but at the cost of penalizing other performance parameters. For example, using the ETX metric may help the routing process to use more reliable paths, however, it may lead to excessive use of some network nodes reducing the remaining energy. Thus, it is necessary to combine several metrics to be able to characterize the route quality more efficiently. For the best route selection, it is proposed the combination of several important routing metrics.

The objective functions proposed in this approach enable the devices to select the parent nodes (default route) based on the context information obtained from the application. Each application may have requirements that simultaneously needs up to three metrics (Expected Transmission Count (ETX), Number of Hops (*NH*), and/or Energy Consumed (*EC*)). These requirements allow rating them according to their priority level (N) considering the following levels: High = 1; Medium = 3; and Low = 5. Each metric is represented by an *F* function. Based on this information, a *T*(*ni*) weight is achieved by each network device, which represents the sum of the context functions.

• Delivery Quality and Context Aware Type 1 Objective Function (DQCA-OF1):

(10) T(ni)= NETX · FETX(ni) + NNH · FNH(ni)

The DQCA-OF1 function considers the metrics Expected Transmission Count (ETX) and Number of Hops (*NH*) for the calculation of the route selection.

• Delivery Quality and Context Aware Type 2 Objective Function (DQCA-OF2):

(11) T(ni)= NETX · FETX(ni) + NEC · FEC(ni) 

The DQCA-OF2 function includes the metrics Expected Transmission Count (ETX) and Energy Consumed (*EC*) for the route selection calculation.

• Delivery Quality and Context Aware Type 3 Objective Function (DQCA-OF3):

(12) T(ni)= NNH · FNH(ni) + NEC · FEC(ni)

The DQCA-OF3 function uses the metrics Number of Hops (*NH*) and Energy Consumed (*EC*) for the route selection calculation.

• Delivery Quality and Context Aware Type 4 Objective Function (DQCA-OF4):

(13) T(ni)=NETX · FETX(ni) + NNS · FNS(ni) + NEC · FEC(ni)

The DQCA-OF4 function includes the metrics Expected Transmission Count (ETX), Number of Hops (*NH*) and Energy Consumed (*EC*) for the route selection calculation.

#### 3.2.2. Fuzzy System-Based Route Classifier

According to [21], several techniques of Computational Intelligence (CI) have been used to solve routing problems in WSNs. Intelligence techniques reinforce the efficiency of routing protocols in decision making to optimize the network performance. In order to decide efficiently on the choice of the best route among those available, fuzzy logic was used to provide a rigorous algebra to deal with inaccurate information, since it is a convenient method of combining conflicting objectives and specialized human knowledge and allowing implementation in low complexity algorithms. 

**Figure 2:**
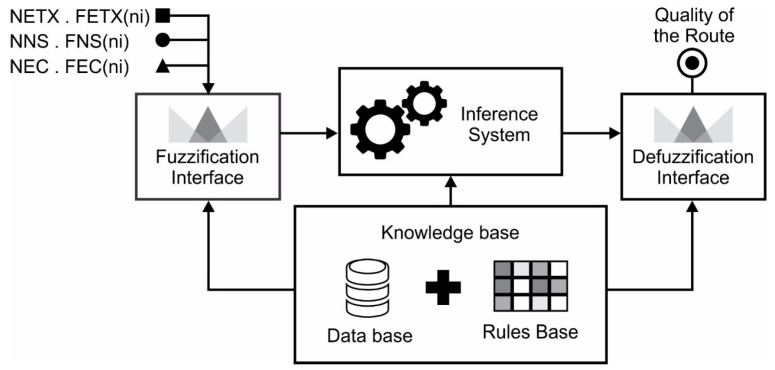
Structure of the Fuzzy System-Based Route Classifier.

To ensure greater consistency in decision making during the routing process, this paper proposes a route classifier based on fuzzy systems, as shown in Figure 2, capable of estimating the degree of quality of the routes, in order to classify them, with the purpose of assisting the selection of routes in IoT scenarios. The route classifier uses as input the values assigned to the metrics (present in the objective functions) specified in Expressions (14)–(16) to rank the routes in order of quality. The trapezoidal form was chosen for the pertinence functions, since it is widely used in fuzzy logic systems [22].

*NETX · FETX* (*ni*) (14)

*NNS · FNS* (*ni*) (15)

*NEC · FEC* (*ni*) (16)

where:

- *NETX* is the ETX priority level;
- *FETX* is the value assigned to the ETX function;
- *NNS* is the priority level of *NS*;
- *FNS* is the value assigned to the *NS* function;
- *NEC* is the priority level of *EC*;
- *FEC* is the value assigned to the *EC* function;
- (*ni*) corresponds to the network node.

The fuzzification interface has the function of mapping the precise inputs to the input fuzzy sets by determining the membership degree, i.e., it transforms quantitative information into qualitative information. Thus, the fuzzification interface can be seen as a function that guarantees a degree of imprecision to a numerical value, mapping the physical value of a process variable into a normalized universe of discourse [22]. For the determination membership degree of each entry, the pertinence functions are used. Several profiles of pertinence functions are found in fuzzy system implementations. The most common and easy to generate pertinence functions are triangular and trapezoidal, although there are other functions such as Gaussian, sigmoid and cubic spline [23].

The knowledge base considers two components, database and rule base. The database contains the primary terms (linguistic terms, natural language terms) for each variable and the membership function of each primary term. The rule base relates (or maps) the system inputs to their outputs, thereby implementing the policies for the estimation, control and decision making of language rules. The rule base performs the mapping of the input domain to the output domain, thus, plays an important role in generating the results produced by the fuzzy inference system.

The inference system is responsible for evaluating the input variables, applying the production rules of the knowledge base and assigning responses to the processing. This response is derived from three steps: evaluation of antecedent, implication and aggregation of the consequent [24]. In the stage of antecedents’ evaluation, the activation of rules occurs, it is in this stage that the degree of pertinence of each proposition present in the antecedent of each rule is calculated. The implication consists in calculating the consequence of the rules that have degree of activation greater than zero. Most of the time, multiple rules are fired at the same time resulting in multiple output sets. Therefore, it is necessary to generate a unique response for each output variable, containing information about all fired rules. The last phase is responsible for aggregating all output sets into a single set. This junction is made according to the fuzzy predicates in the normal form (conjunctive or disjunctive).

Through the use of the route classification system proposed in this work, the messages carry information about expected transmission count, number of hops and energy consumed. This information is used in the fuzzy inference process of the route classifier to determine the quality of the route.

Table 2 shows some fuzzy rules that represent the combination of different metrics, which characterize the input fuzzy sets, i.e., EC, ETX and NS. As the fuzzy system used has three inputs with three pertinence functions for each input, our rule base includes 27 rules, converted to the associated linguistic variables such as Low, Medium and High.

For example, to elect a route with high quality, the energy consumed is low, the number of hops is low and the ETX is low. During the route selection process, fuzzy sets and rules are used according to the requirements of the application.

After the fuzzy inference process, this proposed approach obtains a fuzzy set as an answer. However, because of imprecision, frequently this set is not convenient as a final response of the system, requiring a synthetic numerical representation of the fuzzy response. Thus, function of defuzzification interface is to obtain a precise (non-fuzzy) result in the output of inference system. According to [25], there are several methods of defuzzification highlighting the following: Center of area/gravity; Center of largest area; Average of the maximum; First of the highs; Mean of the highs; and Height of maximum. It was considered in this work a simple and frequently used method, which is the centroid defuzzification method [26] that locates the equilibrium point of the diffuse region of the solution, calculating the weighted average of the diffuse region.

The route selection is based on the quality obtained in the last column of Table 1, which contains a fuzzy output variable, which corresponds to a route quality. From this indicator, the nodes decide locally which route to use to send data without incurring high network costs. 

The rule implemented in the protocol suggests that, after a route is formed, a node must always send data messages using the bigger quality route as observing information contained in your cache.

## 4. Performance Assessment and Results

To evaluate the proposal presented in this work, the COOJA simulator [27] was used to evaluate the performance of networks with devices that run IoT-specific operating systems. In addition, the simulator has an interface to analyze and interact with the nodes, which facilitates the work and network visualization. The COOJA, which is part of the Contiki simulation environment, do not have objective functions that cover all the metrics specified in RFC 6551, being restricted to the metrics of the objective functions adopted by RPL in its original version. For this purpose, the objective functions proposed herein were assessed and compared with the OF0, MRHOF, ERAOF and OF-FL functions. During the simulation experiments, the proposal presented at Ref. [7] was also compared with all simulated proposals in this work. However, the simulation results showed that the proposal of Ref [7] performed bottom when compared to the objective functions evaluated in this work. For this reason, it was not included in the comparative analysis presented in this article. The energy consumption model used for the performance assessment concerning energy, it is the Energest module from the Contiki [28], which measures the energy consumption within an IoT device. 

### 4.1. Network Scenario and Used Metrics

**Figure 3:**
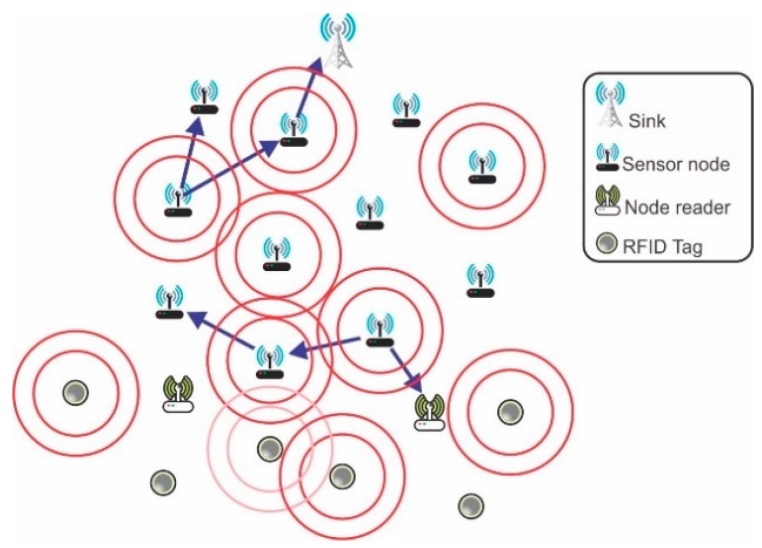
Topology of the network simulation scenario.

The network scenario for simulation was built in order to validate the approach proposed in this paper. It simultaneously includes features from applications aimed at environmental monitoring, tracking and localization. The network topology is shown in Figure 3 and considers 11 sensor nodes, 6 tags (RFID), 2 reader nodes (RFID) and 1 sink node. There are 20 devices within a fixed network topology.

There is data flow for upward and downward. The sink node transmits DODAG Information Object (DIO) messages whenever there is a change of application and it is located as shown in Figure 3.

The use of RFID tags within the simulated scenario is justified because they can play an important role in IoT solutions and it is needed a realistic IoT scenario. According to [29], excess messages exchanged between readers and RFID tags have strong influence on the network’s performance, as well as in application QoS requirements. In the studied scenario, the packets received by the readers are forwarded to the sink, which processes the information sent by readers and by sensor nodes in a consolidated manner.

The total simulation time was 35 min. To ensure that simulations converge to a steady state, each simulation experiment was repeated five times because, after the fifth experiment, no change was observed in the results. However, to evaluate the dynamic change between objective functions, the simulated time was about 140 min, since each application remains for 35 min. The messages were simulated with packets of 30 bytes (Contiki standard). The choice of the parent node for route establishment was based on the Fuzzy System-Based Route Classifier proposed in this work. The initial power of the nodes was adjusted to 200 joules above the Contiki Energest energy consumption model [28]. In this work, the variation in the number of sensor nodes was not considered since, during the study, it was observed that there was no variation of the results changing the number of devices during the use of the proposed objective functions associated to route classifier based on fuzzy system. This is justified because the route selection process of the proposed approach uses only aspects related to energy, position in the routing tree and number of transmission estimates.

The following four metrics used by the RPL (RFC 6551), suitable for 6LoWPAN environments, were chosen for evaluation of this approach: Energy Consumed (*EC*), Number of Hops (*NH*), Delivery Ratio (TX) and Expected Transmission Count (ETX). The Expected Transmission Count (ETX) is equal to the average number of required transmissions (including retransmissions) so that a package is duly delivered to its destination. 

Four applications were considered for this study. The first application requires priority in terms of reliability (represented by the delivery ratio). The second application requires a shorter delay (represented by the number of hops). The third application requires a long lifetime (represented by the energy consumed) and the fourth application requires QoS for data delivery (represented by the ETX). 

### 4.2. Results Analysis

**Figure 4:**
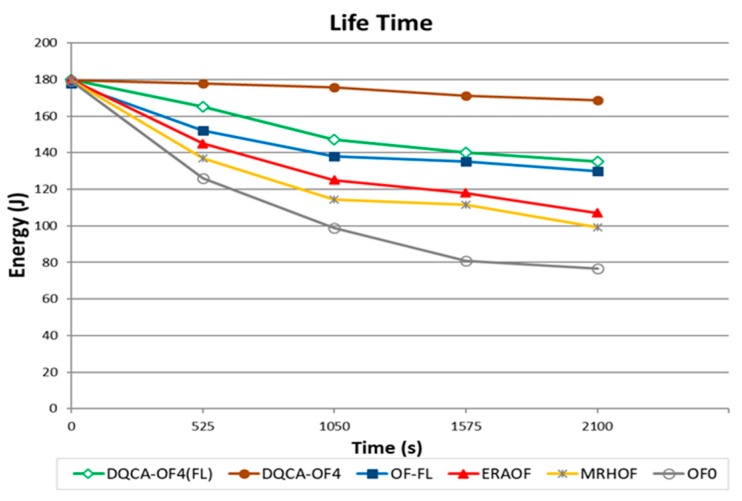
Energy consumption for the network nodes.

The results obtained show that the application that require high priority regarding lifetime and delay used, among the functions available in the DATABASE, the DQCA-OF4 function for the route selection process. This function was selected because it can forecast, in its structure, the metrics energy consumed and number of hops, allowing for the routes selection with lower energy consumed and a lower number of hops. Figure 4 shows the performance of the evaluated objective functions.

The function DQCA-OF4 achieved higher remaining energy (approximately 170 joules), at the end of the simulation, when compared to the DQCA-OF4(FL), OF-FL, ERAOF, MRHOF and OF0 objective functions which achieved consumption rates of 136 joules, 130 joules, 107 joules, 99 joules and 76 joules, respectively. The function DQCA-OF4(FL) obtained lower performance when compared to the DQCA-OF4 as a function of the energy consumed with the complexity of the fuzzy system processing.

**Figure 5:**
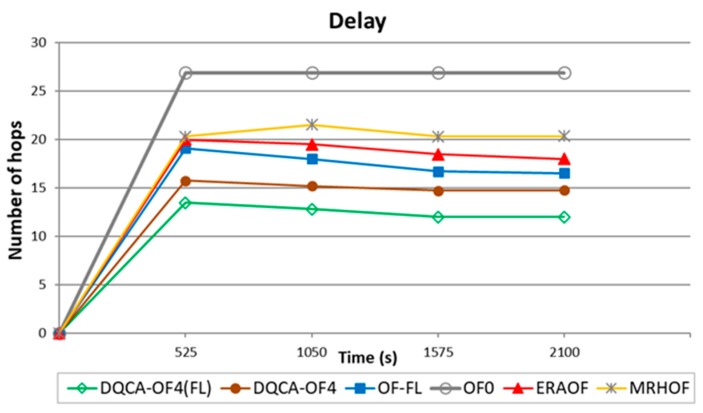
Message delivering delay for the sink.

In Figure 5, from 525 s to the end of the experiment, the DQCA-OF4(FL) function obtained a smaller delay when compared to the DQCA-OF4, OF-FL, ERAOF, MRHOF and OF0 objective functions. This occurred because the DQCA-OF4 (FL) function uses a fuzzy-based route classifier, which is not the case for the objective functions DQCA-OF4, OF-FL, ERAOF, MRHOF and OF0. Figure 5 also shows that there was no variation in results between 0 and 525 s of simulation. This is justified because the network, in this interval, is in the process of stabilization, occurring variation of the delay only after 525 s of simulation.

**Figure 6:**
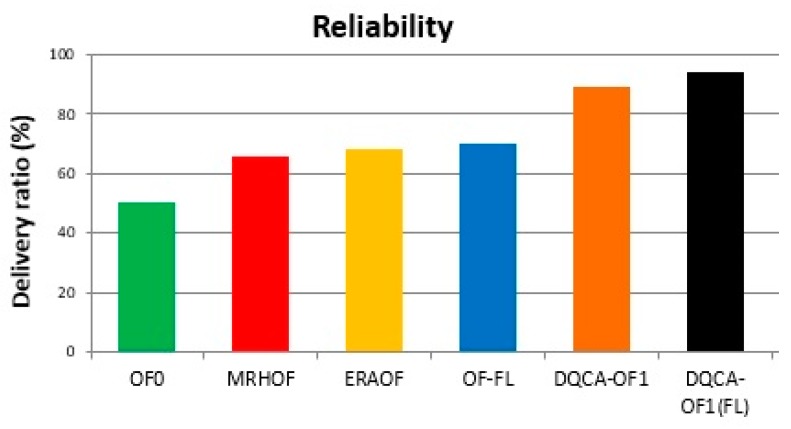
Delivery rate for the sent messages.

For applications that require reliability in the delivery of data, Figure 6 shows that DQCA-OF1(FL) function achieved the best performance, with a delivery ratio of 94% when compared to the DQCA-OF1, OF-FL, ERAOF, MRHOF and OF0 objective functions, which achieved delivery ratios of 89%, 70%, 67.98%, 65.62% and 50.4%, respectively. This was because the expected Transmission Count (ETX) and Hop Number (NH) metrics; in this case, both with high priority, used the fuzzy-based route classifier.

For reliability, between all the DQCA-OF applied to the classifier of routes based on fuzzy system, only the DQCA-OF1 (FL) was the more efficient when compared with the others. That is why only the DQCA-OF1 (FL) was considered for this analysis. The ETX union with *NH*, represented by DQCA-OF1 (FL), demonstrated to be relevant, since the application requiring reliability requires low ETX. In addition, the presence of the number of hops in this function allowed to increase the percentage of reliability, because the smaller the number of hops the greater the probability of delivery of the messages.

**Figure 7:**
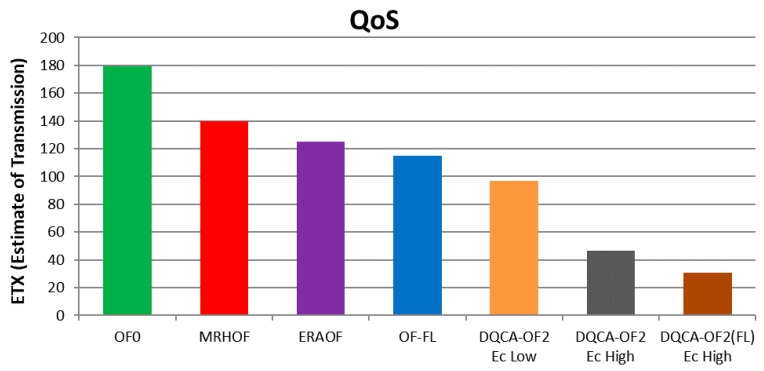
Quality of Service (QoS).

Figure 7 shows that, for applications that require priority in the quality of the delivery of data, the DQCA-OF2(FL) with EC = High achieved the best performance in the number of transmissions/retransmissions represented by ETX. The DQCA-OF2(FL) with *EC* = High got ETX = 31 when compared to the DQCA-OF2 (EC = High) functions, which achieved ETX = 46, DQCA-OF2 (*EC* = Low) with ETX = 96, OF-FL with ETX = 115, ERAOF with ETX = 125, MRHOF with ETX = 140 and OF0 with ETX = 180. This difference was achieved because the application required a high level of priority in the Energy consumed (*EC*) metric combined with the use of route classifier based on fuzzy system.

For the QoS, between all the DQCA-OF applied to the classifier of routes based on fuzzy system, only the DQCA-OF2 (FL) with Ec = high (high priority for energy consumed), was the more efficient when compared with the others. That is why only the DQCA-OF2 (FL) was considered for this analysis. The ETX junction with EC, represented by DQCA-OF2 (FL), proved to be important, since the application requiring QoS requires also low ETX. In addition, the presence of the metric energy consumed in this function, allows the increase of the network lifetime. These two benefits, increase the QoS of the network. 

### 4.3. Statistical Data Analysis

To indicate the dispersion or variability of the data in terms relative to its mean value, it was used as a measure of dispersion the CVP (Pearson Variation Coefficient) with confidence degree of 95%. The CVP was used because it is a relative measure of variability and it is independent of the unit of measure used, where the observed data unit may be different and its value will not change. The CVP is given by the relation between the standard deviation and the mean referring to the data of the same sample.

(17) $$\mathrm{CVP}=\frac{\sigma }{\bar{X}}\cdot 100$$

where:

- CVP is the Pearson Coefficient of Variation
- *σ* is the standard deviation of the series data
- $\bar{X}$ is the average of the series data

For the energy consumed metric, Table 3 shows that DQCA-OF4 presented lower value in the coefficient of variation with 2.15% when compared to DQCA-OF4 with the fuzzy system (8.64%), OF-FL (11.06%), ERAOF (13.89%), MRHOF (19.04%) and OF0 (29.92%). This shows that DQCA-OF4 data, in the metric energy consumed, are more homogeneous when compared to the data of the other objective functions, representing low dispersion around the mean.

For delay metric, Table 4 shows that DQCA-OF4 and DQCA-OF4(FL) presented similar dispersion represented by CVP. It is possible to observe that the four functions have similar stability. However, analyzing the mean, the DQCA-OF4(FL) was more efficient (see the value 11 in Table 4) when compared to the other objective functions. In this case, the lower the average, the lower the delay. The low standard deviation (shown in Table 3 and Table 4) indicates that the data points tend to be close to the average, allowing variability to be expressed between the data.

## 5. Conclusion and Future Work

This paper proposes an approach to the dynamic routes selection for IoT based on context information from the applications. The approach consists the creation of four new Objective Functions (OFs). Each one of these objective functions is based on the fusion of metrics for 6LoWPAN environments, used by the RPL and described in RFC 6551. Each application may have requirements that simultaneously need up to three metrics (Expected Transmission Count (ETX) and/or Number of Hops (*NH*) and/or Energy Consumed (*EC*)). Additionally, the approach also accounts Route Classifier based on Fuzzy System, that estimating the degree of quality of the routes in IoT scenarios. The route classifier uses as input the values assigned to the metrics (present in the objective functions), ensuring consistency in decision making during the routing process. In general, it was observed that, for the scenarios evaluated, the proposed approach can increase the performance of the network to be used by an application of IoT. The approach proved to be able to increase the life time, to reduce the delay and to increase the reliability and the QoS.

It was shown that, using the proposed approach, the lifetime of the network increased and the energy consumption during the operation of the network reduced compared to the other proposals. The approach is more effective to use Objective Functions (OFs) dynamically (at run time) and to choose the path of the high quality by means of fuzzy System-Based Route Classifier. The route with the highest quality is selected as the preferred route. Thus, it is possible to observe that the proposed approach has substantial advantages in relation to the other proposals researched, mainly in the metric time of life, which obtained 168.72 J, allowing the network to function for 2100 s. In addition, provides high reliability by successfully delivering nearly 100% of data packets, low delay for data delivery and increase in QoS. An 30% improvement is attained in the network life time when using one of proposed objective function, keeping the devices alive for longer duration.

Based on the results presented, a specific objective function is recommended for each application. For application that requires a long lifetime, DQCA-OF4 is recommended. For application that requires a shorter delay, DQCA-OF4 (FL) is the more indicated. DQCA-OF1 (FL) is more recommended for applications that prioritize reliability. For application that requires QoS for data delivery, it is suggested to use DQCA-OF2 (FL).

The main contribution of this work, compared to the proposals available in the literature and considered in the study, includes an optimization of the RPL protocol to reduce the delay and to increase the reliability and QoS, guaranteeing also an increase in the network life-time. In this proposal, it is not considered the variation in the number of sensor nodes and RFID tags, the mobility of both the sink and the RFID reader and the network nodes.

Next steps include the deployment of this approach in other routing protocols for IoT, taking into account the scalability of objects that performs an IoT structure. It is also intended to carry out new studies to optimize the objective functions as well as the route classifier.

## Figures and Tables

**Table 1:** Symbols definition.

Symbol	Meaning
*n*	nodes connected to network
*Ns*	set of network nodes
*L*	set links between nodes
*ni*	node enabled to have a parent
*A*(*ni*)	set of progenitor nodes
*ns*	root node
*l*	number of connections of *ni*
*L*(*ni*)	set of connections to candidate nodes
*aj*	node selected as parent
*ap*	node selected as child

**Table 2:** Example of fuzzy rule base used.

Energy Consumed	Number of Hops	ETX	Quality of the Route
Low	Low	Low	High
High	Medium	Low	Medium
High	High	Low	Medium
Medium	Low	High	Medium
High	Medium	Medium	Low
Low	High	Medium	Medium

**Table 3:** Statistical Analysis of Energy Consumption.

Objective Functions	Average (J)	Standard Deviation (J)	Coef. Var. Pearson
DQCA-OF4	174	3	2.15%
DQCA-OF4 (LF)	151	13	8.64%
OF-FL	145	16	11.06%
ERAOF	132	18	13.89%
MRHOF	126	24	19.04%
OF0	109	32	29.92%

**Table 4:** Statistical Analysis of Delay.

Objective Functions	Average (J)	Standard Deviation (J)	Coef. Var. Pearson
DQCA-OF4 (LF)	11	3	33.48%
DQCA-OF4	12	4	34.22%
OF-FL	15	5	33.62%
ERAOF	16	5	34.06%
MRHOF	17	6	33.81%
OF0	23	8	35.58%
